# Uncovering neglected subtypes and zoonotic transmission of Hepatitis E virus (HEV) in Brazil

**DOI:** 10.1186/s12985-023-02047-6

**Published:** 2023-05-02

**Authors:** Debora Regina Lopes dos Santos, Ricardo Durães-Carvalho, Noemi Rovaris Gardinali, Lais Ceschini Machado, Vanessa Salete de Paula, Gabriel da Luz Wallau, Jaqueline Mendes de Oliveira, Lindomar José Pena, Marcelo Alves Pinto, Laura Helena Vega Gonzalez Gil, Edmilson Ferreira de Oliveira-Filho

**Affiliations:** 1 Department of Virology, Aggeu Magalhães Institute, Oswaldo Cruz Foundation (IAM- FIOCRUZ), Recife, Pernambuco Brazil; 2 Veterinary Institute, Federal Rural University of Rio de Janeiro (UFRRJ), Seropédica, Rio de Janeiro, Brazil; 3 São Paulo School of Medicine, Department of Microbiology, Immunology and Parasitology, Federal University of São Paulo (UNIFESP), São Paulo, SP Brazil; 4 Post-Graduate Program in Structural and Functional Biology, UNIFESP, São Paulo, Brazil; 5 Laboratório de Desenvolvimento Tecnológico em Virologia (LADTV) , Instituto Oswaldo Cruz (IOC), Fundação Oswaldo Cruz (Fiocruz), Rio de Janeiro, Brazil; 6 Laboratório de Tecnologia Virológica (LATEV) , Instituto de Tecnologia em Imunobiológicos (Biomanguinhos), Fundação Oswaldo Cruz (Fiocruz), Rio de Janeiro, Brazil; 7 Department of Entomology and Bioinformatic Core, Oswaldo Cruz Foundation-Fiocruz, Recife, PE Brazil; 8 Laboratory of Molecular Virology, Oswaldo Cruz Institute, Oswaldo Cruz Foundation (IOC- FIOCRUZ), Rio de Janeiro, Brazil; 9 Department of Arbovirology, Bernhard Nocht Institute for Tropical Medicine, WHO Collaborating Center for Arbovirus and Hemorrhagic Fever Reference and Research, National Reference Center for Tropical Infectious Diseases, Bernhard-Nocht-Straße 74, 20359 Hamburg, Germany; 10 Institute of Virology, Charité-Universitätsmedizin Berlin, Corporate Member of Freie Universität Berlin, Humboldt-Universität zu Berlin, Charitéplatz 1, 10117 Berlin, Germany

**Keywords:** Hepatitis E, HEV, Zoonotic transmission, Genetic divergence, Subtypes assignment

## Abstract

Hepatitis E virus (HEV) circulation in humans and swine has been extensively studied in South America over the last two decades. Nevertheless, only 2.1% of reported HEV strains are available as complete genome sequences. Therefore, many clinical, epidemiological, and evolutionary aspects of circulating HEV in the continent still need to be clarified. Here, we conducted a retrospective evolutionary analysis of one human case and six swine HEV strains previously reported in northeastern, southern, and southeastern Brazil. We obtained two complete and four nearly complete genomic sequences. Evolutionary analysis comparing the whole genomic and capsid gene sequences revealed high genetic variability. This included the circulation of at least one unrecognized unique South American subtype. Our results corroborate that sequencing the whole capsid gene could be used as an alternative for HEV subtype assignment in the absence of complete genomic sequences. Moreover, our results substantiate the evidence for zoonotic transmission by comparing a larger genomic fragment recovered from the sample of the autochthonous human hepatitis E case. Further studies should continuously investigate HEV genetic diversity and zoonotic transmission of HEV in South America.

**Supplementary Information:**

The online version contains supplementary material available at 10.1186/s12985-023-02047-6.

## Introduction

Hepatitis E is a zoonotic liver disease infecting 20 million people and causing 44,000 deaths yearly [1]. Hepatitis E virus (HEV) has been detected in a wide range of species and is the sole hepatotropic virus with documented zoonotic transmission [2]. HEV members belong to the family *Hepeviridae* which is subdivided into *Orthohepevirinae* and *Parahepevirinae* subfamilies. The *Orthohepevirinae* subfamily is divided into four genera (*Avihepevirus, Chirohepevirus, Paslahepevirus* and *Rocahepevirus*) of which infect mammals and birds, while the *Parahepevirinae* subfamily has the *Piscihepevirus* genus of which infects trout and salmon. The members of the *Paslahepevirus balayani* species are divided into eight HEV genotypes. Genotypes 1–4 are associated with self-limited acute hepatitis with exclusively human-human (HEV-1 and HEV-2), or zoonotic transmission (HEV-3/HEV-4) [3, 4]. Genotypes 5–8 infect wild mammals [5–7] with one single report of HEV-7 in human, so far [8].

HEV subtyping criteria have been debated mainly because of their subjectivity and lack of consistency [9–11]. Although subtyping is not within the remit of the International Committee on Taxonomy of Viruses (ICTV), the ICTV *Hepeviridae* Study Group has tried to develop an agreed upon set of subtype reference sequences to improve clarity for researchers, epidemiologists and clinicians [12]. Currently, viruses in genotype 3 of the species *Paslahepevirus balayani* are classified into 13 recognized subtypes. HEV unassigned sequences can be recognized as new proposed subtypes if at least three complete genomic sequences from epidemiologically unrelated source form a distinct phylogenetic group [13]. The distribution of genotypes and subtypes differs between geographical regions and hosts [13, 14] and may be associated with different clinical outcomes [15, 16]. Although distance-based criteria for assigning new sequences to subtypes can be used, their limits are not clearly established. Therefore, sequences are assigned to subtypes according to phylogenetic position compared to reference sequences [10, 13].

In South America, due to the low number of partial and complete sequences (~ 340 entries compared to ~ 11,000 from Europe) deposited in GenBank, there is a lack of knowledge about HEV genetic variability (Supplementary Table 1). All previously reported sequences obtained clustered into genotype 3 and were detected in swine herds, pork products, human cases, experimental studies in primates or environmental samples. The few partial sequences obtained for Brazilian sequences indicate that they cluster into subtypes 3b, 3c, 3d, 3f, 3h and 3i. However, the absence of complete genomes prevents accurate analysis of HEV genetic diversity in Brazil. Therefore, in this study, we sequenced available HEV positive samples from previous studies to obtain complete genomic sequences.

## Materials and methods

Sera and feces samples were obtained from six positive swine and one human sample previously reported in studies from the northeastern, southeastern, and southern regions in Brazil [17–21]. The study with human sampling was approved by the Fiocruz institutional ethical committee (protocol PO 307/06) [18]. Sample data, including access numbers, hosts, geographical area, genotype and reported subtype are detailed in Table 1. RNA was extracted and purified from stool or serum samples using TRIzolTM Reagent (Invitrogen) following the manufacturer’s recommendations. cDNA was synthesized with the ImProm-II™ Reverse Transcription System (Promega). RT-PCR was performed using the SuperScript™ III One-Step RT-PCR System with Platinum™ Taq DNA Polymerase kit (Invitrogen) following the manufacturer’s instructions. Primers were designed based on template complete genomes of sequences genetically close to the 156 Brazilian partial HEV-3 sequences available in the GenBank.

**Table 1:** Sample origin and subtyping

Sequence ID (Accession number previous)	Host/Region/state	Published Genotype/ Subtype	Subtype full genome	Subtype capsid	Relevant information
PRsw1 (OQ433914)	Pig/South/Paraná	3b	New subtype proposed	New subtype proposed	Related to sequences from chronically infected patient ﻿(MW596896) and swine (MZ969073) from Uruguay
RJsw1 (OQ433915)	Pig/Southeast/ Rio de Janeiro	3b	New subtype proposed	New subtype proposed	Related to sequences from chronically infected patient ﻿(MW596896) and swine (MZ969073) from Uruguay
RJsw2 (OQ433916)	Pig/Southeast/ Rio de Janeiro	3b		3i	
RJsw3 (OQ433917)	Pig/Southeast/ Rio de Janeiro	3b		New subtype proposed	
RJsw4 (OQ433918)	Pig/Southeast/ Rio de Janeiro	3b		New subtype proposed	
RJh1 (OQ446059)	Human/Southeast/Rio de Janeiro	3b			
PEsw1 (MH664124)	Pig/Northeast/ Pernambuco	Related to 3f	3f	3f	

The complete genome of PRsw1 and PEsw1 were obtained by Sanger sequencing, and the other positive samples were also initially sequenced by Sanger method (primers available in Supplementary Table 1). The failed runs were further tentatively sequenced using DNA-based High-throughput sequencing (HTS) using MiSeq (Illumina) [22]. Sequencing libraries were prepared using Nextera XT Library Kit, and sequencing was performed using a paired-end strategy with a V3–150 cycles flow cell. Low-quality sequencing reads were removed using the default parameters of Trimmomatic 0.36 [23]. Assembly was performed using Velvet 1.0.10 [24], default parameters. Sequence data were compared to all HEV genotype 3 complete or nearly complete genomes (genome size > 6.5 kb; n = 747) available in GenBank as of 28.03.2023, including the references for each HEV-3 subtype [12, 13]. Aiming to investigate the amount of unresolved to fully resolved trees, the phylogenetic signal was verified through likelihood mapping analysis of 10,000 random quartets [25]. Then, phylogenetic reconstruction based on Maximum Likelihood was performed using IQ-Tree server (http://iqtree.cibiv.univie.ac.at/), through default settings, using the best-fit substitution model TIM2 + F + R4 with 1000 ultrafast bootstrap replicates.

## Results and discussion

**Fig. 1:**
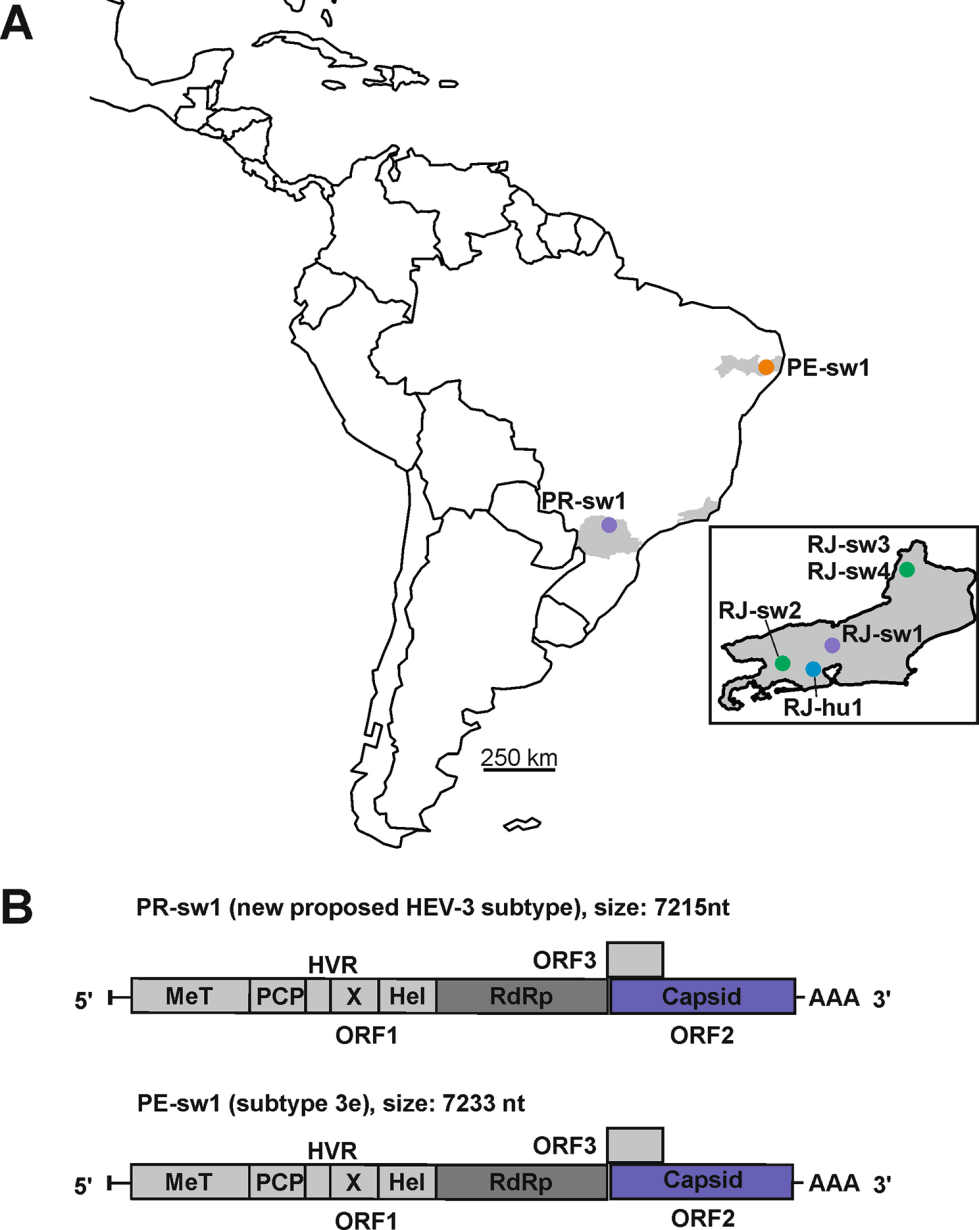
Sample location and HEV genome organization. (**A**) Geographic distribution of HEV sequenced in domestic pigs sampled in different regions from Brazil. Map built with Quantum GIS (http://qgis.osgeo.org). (**B**) Comparison of the genome organization of the two complete sequences obtained in this study. Complete sequence length is given in nucleotides and methyltransferase (Met), a putative papain-like cysteine protease (PCP), hypervariable (HVR) and X domains, RNA helicase (Hel), and the RNA-dependent RNA polymerase (RdRp) are indicated

We were able to sequence two complete genomic sequences (PRsw1 and PEsw1) (Table 1) and, four nearly complete porcine-derived genomic sequence (RJsw1, RJsw2, RJsw3 ad RJsw4), and a fragment of approximately 1000 nucleotides of the first recorded human-derived sequence in Brazil (RJh1). (Fig. 1a). The two complete genomic sequences exhibited the typical features of an HEV genome and were 7215 and 7233 nucleotides length, respectively. Both HEV-3 genomes contain three partially overlapping open reading frames, with ORF3 overlapping ORF1 and ORF2 (Fig. 1b).

**Fig. 2:**
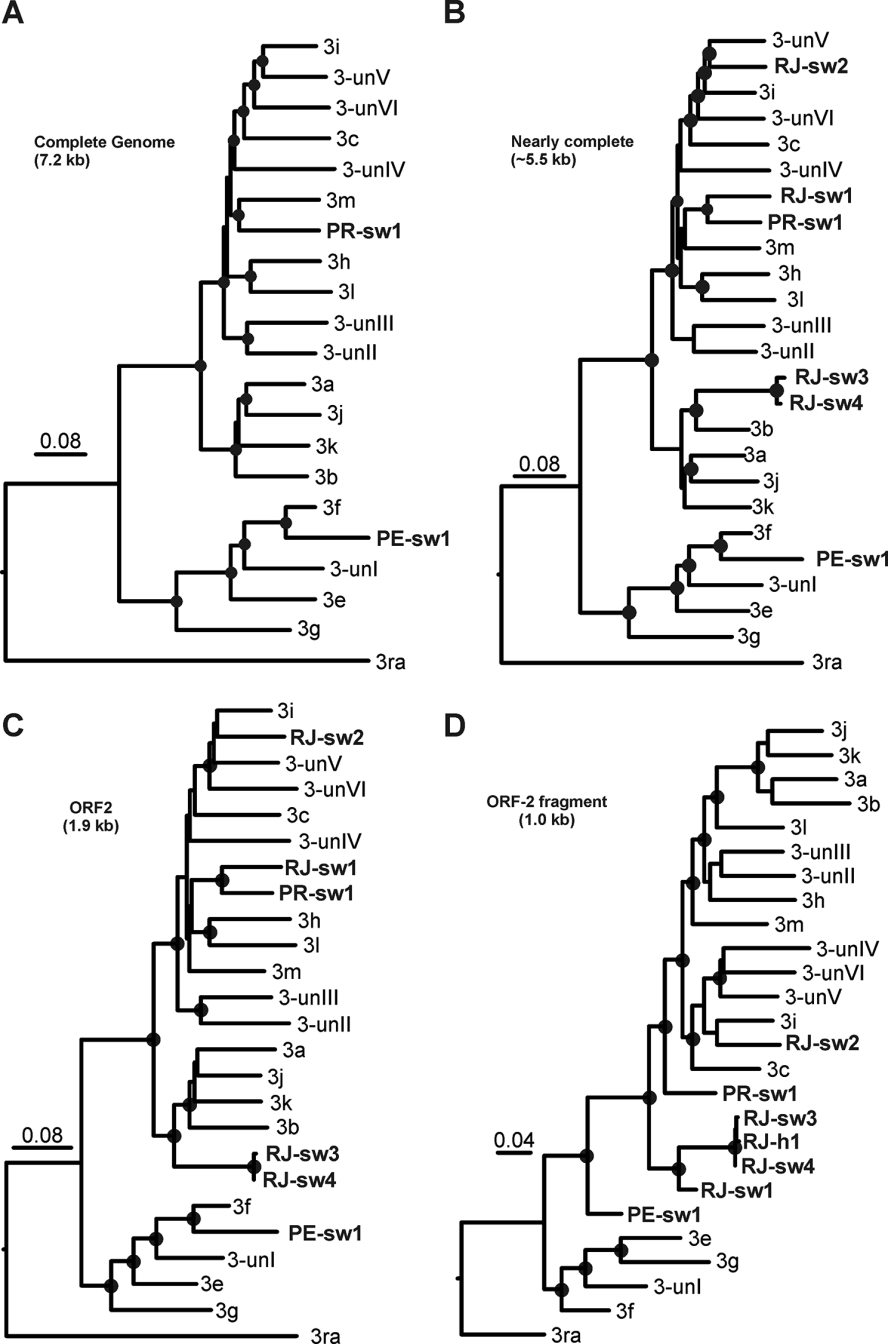
Phylogenetic trees based on nucleotide sequences of complete genomic sequences (**A**) nearly complete (**B**), complete capsid gene (**C**) and partial capsid (**D**) sequences. SH-aLRT/aBayes/ultrafast bootstrap supports values of ≥ 0.80. Detailed information on the evolutionary divergence between our sequences and the reference sequences for each dataset is available on the supplementary Table 2. Reference sequences for HEV-3 subtypes including the remaining unsigned (un) HEV-3 sequences: AF082843 (3a), AP003430 (3b), FJ705359 (3c), AB248521(3e), AB369687(3f), AF455784 (3g), JQ013794 (3h), FJ998008 (3i), AY115488 (3j), AB369689 (3k), JQ953664 (3l ), KU513561(3m), FJ906895 (3ra), AB290313(3-unI), MF959765 (3-unII), LC260517 (3-unIII), MK390971 (3-unIV), MF959764(3-unV), KP294371 (3-unVI)

**Fig. 3:**
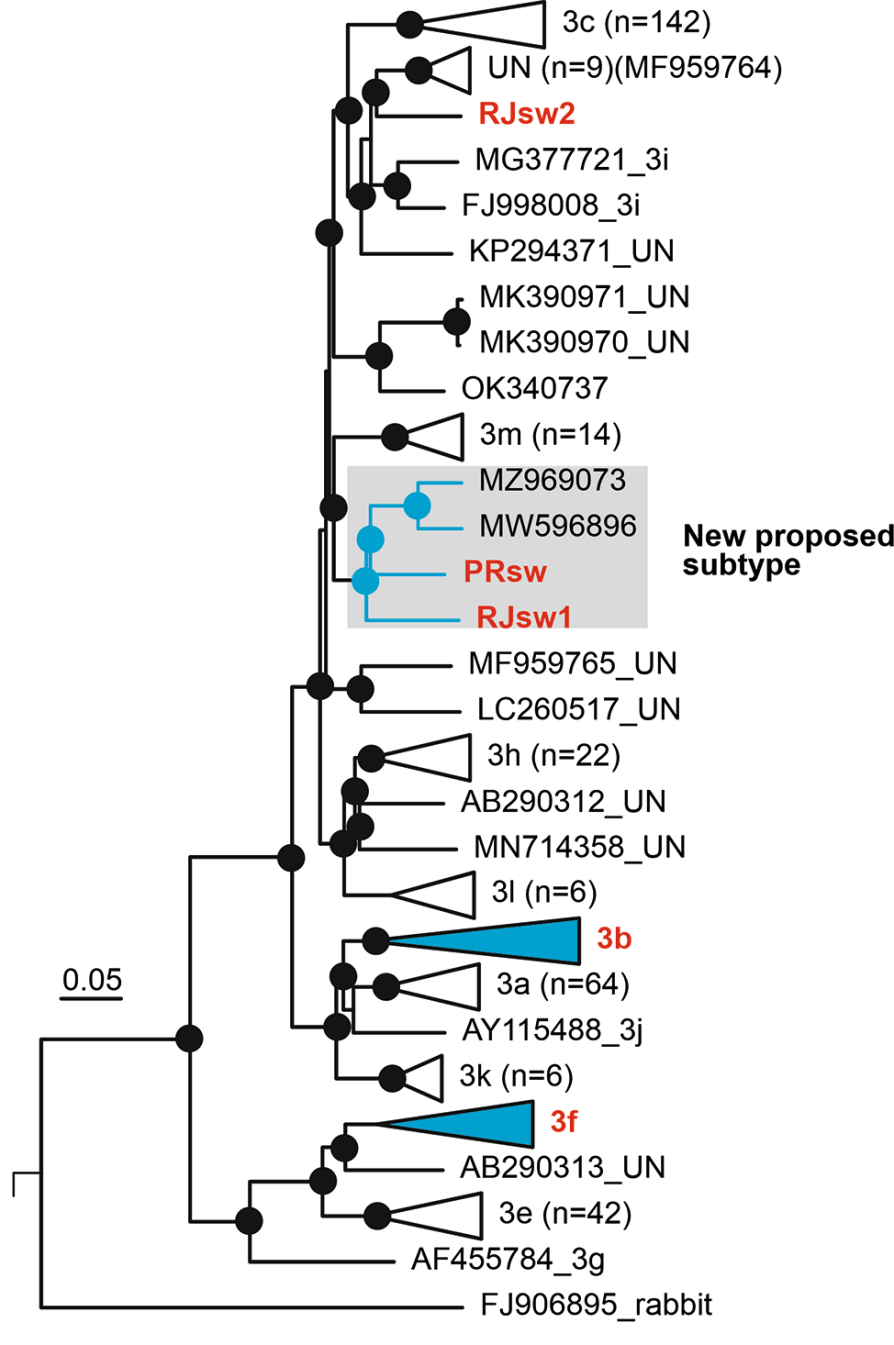
Phylogenetic tree based on nucleotide sequences of nearly complete genomic sequences, including 747 HEV genotype 3 sequences. To improve visualization, subtype branches are collapsed, hence sequences RJsw3 and RJsw4 clustering into subtype 3b and PEsw1 clustering into subtype 3f are omitted. Detailed information, including all sequences used, the alignment, and the complete tree, is available in the supplementary material. SH-aLRT/aBayes/ultrafast bootstrap supports values of ≥ 0.80 were replaced by a circle. Reference sequences for HEV-3 subtypes including the remaining unssined(un) HEV-3 sequences: AF082843 (3a), AP003430 (3b), FJ705359 (3c), AB248521(3e), AB369687(3f), AF455784 (3g), JQ013794 (3h), FJ998008 (3i), AY115488 (3j), AB369689 (3k), JQ953664 (3l), KU513561(3m), FJ906895 (3ra); unassigned sequences are AB290313, MF959765, LC260517, MK390971, MF959764, KP294371

Because we were not able to obtain complete genomic sequences of all positive samples and considering the epidemiological importance of the genetic information, we performed further analyses with multiple datasets to compare with HEV subtype reference sequences. Accordingly, we compared the two complete genomic sequences obtained, the nearly complete genomic sequences and the complete capsid sequence of all six sequences from pigs to a set of HEV-3 reference sequences (Fig. 2a and b). Our analysis suggests that full genome sequences from South (PRsw1) and Southeastern (RJsw1), previously classified as HEV-3 subtype i, likely represent a new subtype with nucleotide divergence of 15.5% from subtype ‘m’ together with two other sequences found in Uruguay (MW596896 and MZ969073) (Fig. 3; Supplementary Table 2). Moreover, the nucleotide divergence of PEsw1 from the northeast region, previously classified as subtype 3f in our previous study [20], differed from the HEV-3f reference sequence at 12.0%, but remained in the same subtype classification. Interestingly, both nearly-complete-genome- and capsid-based analysis suggests that strain RJsw2 (from Southern region) clustered between the subtype 3i and a group of unassigned sequences represented by MF959764 (UN-V). Strains RJsw3 and RJsw4, from the same region, are related to subtype 3b (Fig. 2b). On the one hand, the robust results on the capsid-based phylogeny corroborate that ORF2 sequences can be alternatively used in the absence of full genomic sequences (Fig. 2c), as previously suggested [11, 20, 26], including with subgenomic regions of ORF1 and ORF2 optimized for robust phylogenetic inference and subtyping [26]. By contrast, this illustrates the high genetic variability of the HEV strains circulating in Brazil and upholds the need to increase the number of complete genomic sequences from different samples from Latin America. Overall, the consistent topology derived from multiple inferences supports the robustness of the new proposed subtype.

To investigate the zoonotic origin of the first autochthonous human case of acute hepatitis E reported in Brazil, we compared the partial fragments of the capsid and the ORF1. Phylogenetic analyses using the ORF2 fragment, as well as the previously published ORF1 fragment, indicated that the sequence from the human patient (RJh1) clusters among those from pigs from southeastern region (RJsw3 and RJsw4) with nucleotide identities of 99.5 and 99.6%, respectively (Fig. 2d; Supplementary Fig. 1 and Supplementary Table 2). In the previous study [18], only small fragments of ORF1 and ORF2 were available, and by providing a larger fragment of ORF2, our data support the robustness of the evolutionary inferences and the evidence of zoonotic transmission. Therefore, regardless of whether strains RJsw3 and RJsw4 represent a new subtype or not, our results show strong evidence that the first reported case of HEV in Brazil is likely of zoonotic origin.

Noteworthy, our study is limited by the lack of success in obtaining larger fragments of four sequences which were probably related to the volume availability and conservation status of the samples. However, except for the human sample, the study provided a good amount of retrospective information that allowed robust analysis. Although our findings and those previously published support that the capsid-based analyses are robust, as recommended by the ICTV *Hepeviridae* Study Group, it would be necessary to obtain full genomic sequence before proposing a new subtype [12].

Finally, the genetic variability of HEV in Brazil, along with the occurrence of zoonotic transmission, illustrates the challenges and lack of knowledge of HEV epidemiology in South America. Therefore, further studies, including more representative sequences, are needed to investigate the genetic diversity of HEV in humans, animal reservoirs, food, and environmental samples to obtain an accurate picture of the genetic variability of HEV in South America and its implication in zoonotic transmission and pathogenicity.

## Electronic supplementary material

Below is the link to the electronic supplementary material.

Supplementary Material 1

Supplementary Material 2

Supplementary Material 3

## Data Availability

The raw data supporting the conclusions in this paper will be made available by the authors without reservation.

## References

[1] Hepatitis E. [https://www.who.int/news-room/fact-sheets/detail/hepatitis-e.

[2] Nan Y, Wu C, Zhao Q, Zhou EM (2017). Zoonotic Hepatitis E virus: an ignored risk for Public Health. Front Microbiol.

[3] Kenney SP. The Current Host Range of Hepatitis E Viruses. Viruses 2019, 11. 10.3390/v11050452 PMC6563279 31108942

[4] Purdy MA, Drexler JF, Meng XJ, Norder H, Okamoto H, Van der Poel WHM, Reuter G, de Souza WM, Ulrich RG, Smith DB. ICTV Virus Taxonomy Profile: Hepeviridae 2022. J Gen Virol 2022, 103. 10.1099/jgv.0.001778 PMC12642825 36170152

[5] Takahashi M, Nishizawa T, Nagashima S, Jirintai S, Kawakami M, Sonoda Y, Suzuki T, Yamamoto S, Shigemoto K, Ashida K (2014). Molecular characterization of a novel hepatitis E virus (HEV) strain obtained from a wild boar in Japan that is highly divergent from the previously recognized HEV strains. Virus Res.

[6] Woo PC, Lau SK, Teng JL, Tsang AK, Joseph M, Wong EY, Tang Y, Sivakumar S, Xie J, Bai R (2014). New hepatitis E virus genotype in camels, the Middle East. Emerg Infect Dis.

[7] Woo PC, Lau SK, Teng JL, Cao KY, Wernery U, Schountz T, Chiu TH, Tsang AK, Wong PC, Wong EY, Yuen KY (2016). New Hepatitis E virus genotype in bactrian camels, Xinjiang, China, 2013. Emerg Infect Dis.

[8] Lee GH, Tan BH, Teo EC, Lim SG, Dan YY, Wee A, Aw PP, Zhu Y, Hibberd ML, Tan CK (2016). Chronic infection with Camelid Hepatitis E Virus in a liver transplant recipient who regularly consumes Camel meat and milk. Gastroenterology.

[9] Wang S, Wei W, Luo X, Cai X (2014). Genome-wide comparisons of phylogenetic similarities between partial genomic regions and the full-length genome in Hepatitis E virus genotyping. PLoS ONE.

[10] Nicot F, Dimeglio C, Migueres M, Jeanne N, Latour J, Abravanel F, Ranger N, Harter A, Dubois M, Lameiras S (2020). Classification of the zoonotic Hepatitis E virus genotype 3 into distinct subgenotypes. Front Microbiol.

[11] Oliveira-Filho EF, Konig M, Thiel HJ (2013). Genetic variability of HEV isolates: inconsistencies of current classification. Vet Microbiol.

[12] Smith DB, Simmonds P, Izopet J, Oliveira-Filho EF, Ulrich RG, Johne R, Koenig M, Jameel S, Harrison TJ, Meng XJ (2016). Proposed reference sequences for hepatitis E virus subtypes. J Gen Virol.

[13] Smith DB, Izopet J, Nicot F, Simmonds P, Jameel S, Meng XJ, Norder H, Okamoto H, van der Poel WHM, Reuter G, Purdy MA (2020). Update: proposed reference sequences for subtypes of hepatitis E virus (species Orthohepevirus A). J Gen Virol.

[14] De Sabato L, Suffredini E, Pasquale SD, La Rosa G, De Santis P, Giammarioli M, Vaccari G, Bartolo ID (2022). Novel subtypes and unexpected heterogeneity of hepatitis E viral strains in wild boar captured in a small area in Central Italy. Transbound Emerg Dis.

[15] Peeters M, Schenk J, De Somer T, Roskams T, Locus T, Klamer S, Subissi L, Suin V, Delwaide J, Starkel P (2023). Viral clade is associated with severity of symptomatic genotype 3 hepatitis E virus infections in Belgium, 2010–2018. J Hepatol.

[16] Abravanel F, Dimeglio C, Castanier M, Peron JM, Kamar N, Lhomme S, Izopet J (2020). Does HEV-3 subtype play a role in the severity of acute hepatitis E?. Liver Int.

[17] dos Santos DR, Vitral CL, de Paula VS, Marchevsky RS, Lopes JF, Gaspar AM, Saddi TM, Júnior NC, Guimarães FeR, Júnior JG (2009). Serological and molecular evidence of hepatitis E virus in swine in Brazil. Vet J.

[18] Lopes Dos Santos DR, Lewis-Ximenez LL, da Silva MF, de Sousa PS, Gaspar AM, Pinto MA (2010). First report of a human autochthonous hepatitis E virus infection in Brazil. J Clin Virol.

[19] dos Santos DR, de Paula VS, de Oliveira JM, Marchevsky RS, Pinto MA (2011). Hepatitis E virus in swine and effluent samples from slaughterhouses in Brazil. Vet Microbiol.

[20] Oliveira-Filho EF, Dos Santos DR, Duraes-Carvalho R, da Silva A, de Lima GB, Batista Filho AFB, Pena LJ, Gil LH (2019). Evolutionary study of potentially zoonotic hepatitis E virus genotype 3 from swine in Northeast Brazil. Mem Inst Oswaldo Cruz.

[21] Gardinali NR, Barry AF, da Silva PF, de Souza C, Alfieri AF, Alfieri AA (2012). Molecular detection and characterization of hepatitis E virus in naturally infected pigs from brazilian herds. Res Vet Sci.

[22] Sanger F (1981). Determination of nucleotide sequences in DNA. Science.

[23] Bolger AM, Lohse M, Usadel B (2014). Trimmomatic: a flexible trimmer for Illumina sequence data. Bioinformatics.

[24] Zerbino DR. Using the Velvet de novo assembler for short-read sequencing technologies. *Curr Protoc Bioinformatics* 2010, Chap. 11:Unit 11 15. 10.1002/0471250953.bi1105s31 PMC2952100 20836074

[25] Schmidt HA, Strimmer K, Vingron M, von Haeseler A (2002). TREE-PUZZLE: maximum likelihood phylogenetic analysis using quartets and parallel computing. Bioinformatics.

[26] Purdy MA, Sue A (2017). The effect of phylogenetic signal reduction on genotyping of hepatitis E viruses of the species Orthohepevirus A. Arch Virol.

