# Supplementary material for: Uncovering neglected subtypes and zoonotic transmission of Hepatitis E virus (HEV) in Brazil
Source: Virol J. 2023 May 2;20:83. doi: 10.1186/s12985-023-02047-6 (PMC10152778; doi:10.1186/s12985-023-02047-6)
Supplement: Supplementary file 1 — Supplementary Material 1 [file 12985_2023_2047_MOESM1_ESM.docx]

**Appendix**


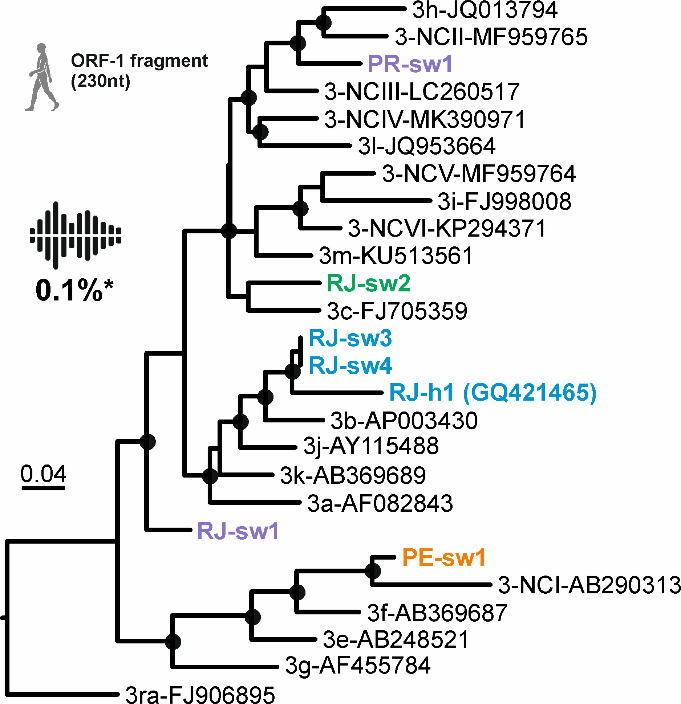


**Supplementary Figure 1.** Phylogenetic trees based on 0.2 kb nucleotide sequences of the ORF1. Sequences obtained in this study is in bold. SH-aLRT/aBayes/ultrafast bootstrap supports values of ≥0.80. Likelihood mapping showing the phylogenetic noise of only 0.1%*. Reference sequences for HEV-3 subtypes including the remaining unclassified (un): AF082843 (3a), AP003430 (3b), FJ705359 (3c), AB248521(3e), AB369687(3f), AF455784 (3g), JQ013794 (3h), FJ998008 (3i), AY115488 (3j), AB369689 (3k), JQ953664 (3l), KU513561(3m), AB290313(3-unI), MF959765 (3-unII), LC260517 (3-unIII), MK390971 (3-unIV), MF959764(3-unV), KP294371 (3-unVI).

**Supplementary Table 1** Number of HEV entries on the GenBank per continent (June 2022)

| **Region** | **Number** | **% of full genome** |
| --- | --- | --- |
| Africa | 320 | 1.6% |
| South America | 340 | 2.1% |
| Europe | 11301 | 4.1% |
| North America | 703 | 5.4% |
| Asia | 8951 | 6.3% |
| Oceania | 156 | 17.9% |

**Supplementary Table 2.** Primers used for sequencing complete genomic sequences

| **Names** | **Oligonucleotides** | **Reference** |
| --- | --- | --- |
| pORF1-3F | ATGGAGGCCCATCAGTTCATTAA | Adapted from [1] |
| pORF1-3R | AGCATGAGCCGATCCCA | Adapted from [1] |
| ORF1_1F | AGACCACGTATGTGGTCGAT | Adapted from [1] |
| ORF1_2F | GCCTGTACGCTGCACTACA | Adapted from [1] |
| ORF1_3F | TTCCTTACCCTCGATCGACA | Adapted from [1] |
| ORF1_4R | AGGTGTACGTCCGCTCTATA | Adapted from [1] |
| ORF1_5R | TGTAGAGCAAGCTGACGGAA | Adapted from [1] |
| ORF1_6R | TGTAGATTTTGTAGAGCAAGCTG | Adapted from [1] |
| ORF1_7R | TGATAACCAACGGCGACATTG | Adapted from [1] |
| ORF1_8R | TTCTTAAGAAACGTCCTGCAAC | Adapted from [1] |
| ORF1_9R | TGACCTTGATCGCCAACCAA | Adapted from [1] |
| ORF1_10F | CAGAAATTCATCACAAGACTCTA | Adapted from [1] |
| ORF1_11F | TCAGCTCCAGTTTTATGCACAA | Adapted from [1] |
| ORF1_12F | CCAGCTCGAGGGTCTGTA | Adapted from [1] |
| ORF1_13F | GCACTCAATATCCCGCATGA | Adapted from [1] |
| ORF1_14R | GTAGCAGTCAGACGAGACG | Adapted from [1] |
| ORF1_15R | TGATGAACTCAGTCGGATGAA | Adapted from [1] |
| ORF1_16R | TTGTGCTCGACCCTATAATCG | Adapted from [1] |
| ORF1_17R | TTCTCGGTACGCTGCCTCA | Adapted from [1] |
| pORF1-4F | AAGGTGTATGCGGGGTCATTG | Adapted from [1] |
| pORF1-4R | AGCTCACACACATCAGCCGG | Adapted from [1] |
| pORF1-2F | TGGTGGCACGTTACACACC | Adapted from [1] |
| pORF1-2R | TCTCCACCATGGCCTCAAC | Adapted from [1] |
| pORF1HEV-1F | ATGGTGGAGAAGGGHCAGGA | Adapted from [1] |
| pORF1HEV-1R | ATCAACACAAACCTGCGCAACA | Adapted from [1] |
| Cap1_Ext__F | GCGCAGGTYTGTGTTGATGT | Adapted from [2] |
| Cap1_Ext_R | TACTGGGCATRGTTRGAYGCCTC | Adapted from [2] |
| Cap1_Int_F | GGGYTGGTRCATAACCTYATTGG | Adapted from [2] |
| Cap1_Int_R | GCCATAATRTGTGTRTTGGTGCC | Adapted from [2] |
| Cap2_Ext_F | TCACCGGCCCCYGAYAC | Adapted from [2] |
| Cap2_Ext_R | ARSCGRTGGCGGGCTGT | Adapted from [2] |
| Cap2_Int_F | TGCGACGACAGTATAAYYT | Adapted from [2] |
| Cap2_Int_R | GTRTACCGRGATACACG | Adapted from [2] |
| Cap3_Ext_F | TGGTGATGCTYTGYATTCATGG | Adapted from [2] |
| Cap3_Ext_R | ACCARTCMAGAGARCGGG | Adapted from [2] |
| CAP3_Int_F | CTTGAYTTYGCGYTAGARCTTGA | Adapted from [2] |
| Cap3_Int_R | CCTGRGCCCCTGTTGCYA | Adapted from [2] |
| Cap4_Ext_F | GAGTAYGAYCAGACTACGTATGG | Adapted from [2] |
| Cap4_Int_F | TCCACCAACCCGATGTATGT | Adapted from [2] |
| 15T-aTag | CCAACGACCGGGAGGCCATTTTTTTTTTTTTTTV | Adapted from [2] |
| TAG | CCAACGACCGGGAGGCCA | Adapted from [2, 3] |

**Supplementary Table 3** Estimates pairwise distance of multiple sequence dataset between sequences reported in this study and HEV genotype 3 reference

|  | **3a** | | | | | **3b** | | | | | **3c** | | | | | **3e** | | | | | **3f** | | | | | **3g** | | | | |
| --- | --- | --- | --- | --- | --- | --- | --- | --- | --- | --- | --- | --- | --- | --- | --- | --- | --- | --- | --- | --- | --- | --- | --- | --- | --- | --- | --- | --- | --- | --- |
| Dataset: | **Co** | **A** | **Cp** | **H1** | **H2** | **Co** | **A** | **Cp** | **H1** | **H2** | **Co** | **A** | **Cp** | **H1** | **H2** | **Co** | **A** | **Cp** | **H1** | **H2** | **Co** | **A** | **Cp** | **H1** | **H2** | **Co** | **A** | **Cp** | **H1** | **H2** |
| PR-sw1 | 17.5 | 16.9 | 17.4 | 18.1 | 18.7 | 17.6 | 16.9 | 15.9 | 13.9 | 17.2 | 16.1 | 15.4 | 14.5 | 14.8 | 13.6 | 23.2 | 21.4 | 17.6 | 26.9 | 19.5 | 22.6 | 21.3 | 17.7 | 25.3 | 19.7 | 22.3 | 21.2 | 17.8 | 21.3 | 19.5 |
| PE-sw1 | 23.2 | 22.4 | 19.1 | 25.8 | 19.8 | 23.6 | 22.7 | 17.9 | 26.4 | 19.1 | 22.3 | 21.1 | 18.2 | 23.6 | 18.7 | 18.2 | 17.2 | 14.6 | 15.0 | 17.4 | **12.0** | **12.4** | **10.7** | **14.4** | **12.7** | 21.3 | 21.0 | 17.2 | 21.3 | 19.6 |
| RJ-sw1 | - | 17.9 | 16.7 | 18.8 | 18.5 | - | 17.9 | 16.4 | 16.1 | 17.1 | - | 16.1 | 14.1 | 14.2 | 14.4 | - | 21.7 | 17.9 | 19.6 | 19.5 | - | 21.6 | 18.8 | 20.2 | 21.5 | - | 20.9 | 18.2 | 19.3 | 21.8 |
| RJ-sw2 | - | 17.4 | 16.9 | 17.2 | 18.0 | - | 17.4 | 16.4 | 19.2 | 17.6 | - | 15.1 | 14.8 | 13.0 | 16.4 | - | 21.4 | 19.1 | 25.4 | 21.7 | - | 21.9 | 19.1 | 24.1 | 21.9 | - | 21.2 | 17.8 | 19.1 | 21.1 |
| RJ-sw3 | - | 15.5 | 13.7 | 15.2 | 15.7 | - | 14.7 | 12.9 | 8.7 | 15.1 | - | 18.6 | 15.9 | 16.8 | 15.8 | - | 20.8 | 17.1 | 25.0 | 19.3 | - | 20.5 | 18.2 | 25.8 | 20.5 | - | 21.6 | 18.4 | 21.8 | 21.7 |
| RJ-sw4 | - | 15.0 | 13.4 | 15.2 | 15.4 | - | 14.1 | 12.5 | 8.7 | 14.6 | - | 18.4 | 15.6 | 16.8 | 15.4 | - | 20.8 | 16.7 | 25.0 | 19.0 | - | 20.8 | 17.9 | 25.8 | 20.2 | - | 21.5 | 17.9 | 21.8 | 21.2 |
| RJ-h1 | - | - | - | 19.6 | 15.6 | - | - | - | 12.7 | 17.9 | - | - | - | 17.3 | 15.4 | - | - | - | 26.3 | 18.9 | - | - | - | 26.0 | 19.9 | - | - | - | 24.9 | 21.3 |
|  |  |  |  |  |  |  |  |  |  |  |  |  |  |  |  |  |  |  |  |  |  |  |  |  |  |  |  |  |  |  |
|  | **3h** | | | | | **3i** | | | | | **3j** | | | | | **3k** | | | | | **3l** | | | | | **3m** | | | | |
| Dataset: | **Co** | **A** | **Cp** | **H1** | **H2** | **Co** | **A** | **Cp** | **H1** | **H2** | **Co** | **A** | **Cp** | **H1** | **H2** | **Co** | **A** | **Cp** | **H1** | **H2** | **Co** | **A** | **Cp** | **H1** | **H2** | **Co** | **A** | **Cp** | **H1** | **H2** |
| PR-sw1 | 16.3 | 15.7 | 14.1 | 14.1 | 14.5 | 15.9 | 14.9 | 13.4 | 18.7 | 13.4 | 19.0 | 18.4 | 16.4 | 17.1 | 16.4 | 17.3 | 16.6 | 15.7 |  | 18.2 | 16.5 | 16.1 | 14.3 |  | 15.1 | 15.5 | 14.6 | 13.0 |  | 14.2 |
| PE-sw1 | 22.8 | 21.5 | 17.6 | 25.8 | 18.4 | 22.4 | 21.0 | 17.3 | 21.1 | 18.6 | 23.3 | 22.1 | 19.1 | 23.7 | 17.3 | 23.9 | 22.5 | 17.6 |  | 19.3 | 22.1 | 20.7 | 18.2 |  | 20.5 | 23.9 | 22.6 | 18.6 |  | 19.4 |
| RJ-sw1 | - | 16.1 | 14.2 | 20.4 | 16.0 | - | 14.9 | 13.6 | 17.8 | 15.1 | - | 18.1 | 14.0 | 15.0 | 16.1 | - | 18.0 | 16.4 |  | 18.4 | - | 16.9 | 15.1 |  | 16.9 | - | 15.8 | 13.3 |  | 15.6 |
| RJ-sw2 | - | 16.3 | 12.7 | 21.6 | 16.5 | - | **12.3** | **10.9** | **9.4** | **12.4** | - | 17.9 | 17.7 | 16.6 | 19.6 | - | 17.4 | 16.0 |  | 17.8 | - | 15.5 | 13.9 |  | 15.7 | - | 15.3 | 13.6 |  | 15.8 |
| RJ-sw3 | - | 18.1 | 14.9 | 21.1 | 16.5 | - | 18.0 | 14.6 | 20.1 | 15.9 | - | 16.1 | 18.4 | 9.8 | 15.1 | - | 16.6 | 13.7 |  | 16.1 | - | 18.8 | 15.9 |  | 15.2 | - | 18.4 | 15.3 |  | 15.9 |
| RJ-sw4 | - | 17.9 | 14.8 | 21.1 | 16.7 | - | 17.5 | 14.3 | 20.1 | 15.5 | - | 15.7 | 18.3 | 9.8 | 14.6 | - | 16.3 | 14.7 |  | 15.9 | - | 18.6 | 15.7 |  | 15.1 | - | 18.0 | 15.1 |  | 15.8 |
| RJ-h1 | - | - | - | 18.9 | 16.5 | - | - | - | 20.2 | 15.8 | - | - | - | 14.3 | 15.0 | - | - | - |  | 16.2 | - | - | - |  | 15.1 | - | - | - |  | 15.8 |
|  |  |  |  |  |  |  |  |  |  |  |  |  |  |  |  |  |  |  |  |  |  |  |  |  |  |  |  |  |  |  |
|  | **3-NCI-AB290313** | | | | | **3-NCII-MF959765** | | | | | **3-NCIII-LC260517** | | | | | **3-NCIV-MK390971** | | | | | **3-NCV-MF959764** | | | | | **3-NCVI-KP294371** | | | | |
| Dataset: | **Co** | **A** | **Cp** | **H1** | **H2** | **Co** | **A** | **Cp** | **H1** | **H2** | **Co** | **A** | **Cp** | **H1** | **H2** | **Co** | **A** | **Cp** | **H1** | **H2** | **Co** | **A** | **Cp** | **H1** | **H2** | **Co** | **A** | **Cp** | **H1** | **H2** |
| PR-sw1 | 22.8 | 21.4 | 18.9 |  | 21.5 | 16.0 | 15.4 | 14.3 |  | 14.5 | 16.9 | 16.0 | 13.5 |  | 14.2 | 17.0 | 16.0 | 13.9 |  | 15.8 | 16.0 | 15.0 | 13.7 |  | 13.4 | 16.3 | 15.4 | 13.6 |  | 14.8 |
| PE-sw1 | 18.0 | 17.6 | 14.7 |  | 19.1 | 23.1 | 21.9 | 19.0 |  | 18.9 | 23.3 | 21.8 | 17.0 |  | 16.6 | 23.1 | 21.1 | 18.0 |  | 18.9 | 22.0 | 20.9 | 17.1 |  | 17.8 | 22.9 | 21.5 | 18.1 |  | 19.1 |
| RJ-sw1 | - | 21.8 | 18.3 |  | 20.0 | - | 16.5 | 15.4 |  | 17.2 | - | 16.1 | 13.9 |  | 14.7 | - | 17.1 | 15.2 |  | 17.6 | - | 16.3 | 14.3 |  | 15.6 | - | 15.5 | 14.5 |  | 15.9 |
| RJ-sw2 | - | 21.5 | 18.1 |  | 20.3 | - | 15.5 | 15.4 |  | 17.7 | - | 16.2 | 14.8 |  | 15.1 | - | 16.2 | 14.6 |  | 15.5 | - | 12.4 | 11.9 |  | 14.2 | - | 14.1 | 12.7 |  | 14.0 |
| RJ-sw3 | - | 21.6 | 18.7 |  | 19.6 | - | 18.5 | 16.7 |  | 17.6 | - | 18.6 | 14.2 |  | 15.2 | - | 19.3 | 16.2 |  | 18.4 | - | 17.7 | 14.8 |  | 15.7 | - | 18.4 | 15.5 |  | 17.6 |
| RJ-sw4 | - | 21.7 | 18.4 |  | 19.4 | - | 18.1 | 16.4 |  | 17.5 | - | 17.9 | 13.9 |  | 15.1 | - | 19.0 | 16.1 |  | 18.3 | - | 17.6 | 14.6 |  | 15.5 | - | 18.3 | 15.3 |  | 17.6 |
| RJ-h1 | - | - | - |  | 19.1 | - | - | - |  | 17.5 | - | - | - |  | 15.1 | - | - | - |  | 18.8 | - | - | - |  | 15.8 | - | - | - |  | 18.0 |

* Nucleotide pairwise sequence distance between sequences is shown. Abbreviations: A: Alignment with all sequences without gaps (5713), Cp: Alignment of the capsid region with all sequences (1986), Co: Alignment with the two complete genomes (7162), H1: Alignment with the human genome region ORF 1 (242), H2: Alignment with the human genome region ORF 2 (1033).

**Supplementary Table 4.** Position and size of gaps in the HEV sequences

| **Sequence ID** | **Gap position** | **Size** |
| --- | --- | --- |
| RJ-sw3 (OQ433917) | 1794-2387 | 594 |
|  | 4903-5041 | 139 |
| RJ-sw4 (OQ433918) | 1801-1820 | 20 |
|  | 1895-2246 | 352 |
|  | 2321-2389 | 69 |
| RJ-sw2 (OQ433916) | 948 - 1531 | 584 |
| RJ-sw1 (OQ433915) | 5004-5037 | 34 |

**References**

1. Oliveira-Filho EF, Bank-Wolf BR, Thiel HJ, König M: **Phylogenetic analysis of hepatitis E virus in domestic swine and wild boar in Germany.** *Vet Microbiol* 2014.

2. Oliveira-Filho EF, Dos Santos DR, Duraes-Carvalho R, da Silva A, de Lima GB, Batista Filho AFB, Pena LJ, Gil LH: **Evolutionary study of potentially zoonotic hepatitis E virus genotype 3 from swine in Northeast Brazil.** *Mem Inst Oswaldo Cruz* 2019, **114:**e180585.

3. Muller B, Klemm U, Mas Marques A, Schreier E: **Genetic diversity and recombination of murine noroviruses in immunocompromised mice.** *Arch Virol* 2007, **152:**1709-1719.
